# Phylogeny and biogeography of the amphi-Pacific genus *Aphananthe*

**DOI:** 10.1371/journal.pone.0171405

**Published:** 2017-02-07

**Authors:** Mei-Qing Yang, De-Zhu Li, Jun Wen, Ting-Shuang Yi

**Affiliations:** 1 Germplasm Bank of Wild Species, Kunming Institute of Botany, Chinese Academy of Sciences, Kunming, Yunnan, China; 2 Baotou Medical College, Baotou, Inner Mongolia, China; 3 Department of Botany, National Museum of Natural History, Smithsonian Institution, Washington DC, United States of America; Institute of Botany, CHINA

## Abstract

*Aphananthe* is a small genus of five species showing an intriguing amphi-Pacific distribution in eastern, southern and southeastern Asia, Australia, and Mexico, also with one species in Madagascar. The phylogenetic relationships of *Aphananthe* were reconstructed with two nuclear (ITS & ETS) and two plastid (*psbA*-*trnH* & *trnL*-*trnF*) regions. Clade divergence times were estimated with a Bayesian approach, and the ancestral areas were inferred using the dispersal-extinction-cladogenesis and Bayesian Binary MCMC analyses. *Aphananthe* was supported to be monophyletic, with the eastern Asian *A*. *aspera* resolved as sister to a clade of the remaining four species. *Aphananthe* was inferred to have originated in the Late Cretaceous (71.5 mya, with 95% HPD: 66.6–81.3 mya), and the crown age of the genus was dated to be in the early Miocene (19.1 mya, with 95% HPD: 12.4–28.9 mya). The fossil record indicates that *Aphananthe* was present in the high latitude thermophilic forests in the early Tertiary, and experienced extinctions from the middle Tertiary onwards. *Aphananthe* originated in Europe based on the inference that included fossil and extant species, but eastern Asia was estimated to be the ancestral area of the clade of the extant species of *Aphananthe*. Both the West Gondwanan vicariance hypothesis and the boreotropics hypothesis could be excluded as explanation for its amphi-Pacific distribution. Long-distance dispersals out of eastern Asia into North America, southern and southeastern Asia and Australia, and Madagascar during the Miocene account for its wide intercontinental disjunct distribution.

## Introduction

The amphi-Pacific tropical disjunction has long been discussed as an important biogeographic pattern in plants, with more than 100 genera and higher taxa of angiosperms exhibiting this distribution pattern [1–2]. Two major hypotheses have been proposed to explain amphi-Pacific tropical disjunctions. The boreotropics hypothesis postulates a continuous belt of tropical to subtropical forest at middle to northern latitudes of the Northern Hemisphere, and the continents were connected by the Bering and North Atlantic land bridges during the early Cenozoic [3–7]. Thermophilic taxa may have migrated across continents through two land bridges, especially the North Atlantic land bridges, until the late Eocene [4–5, 8]. The subsequent gradually cooling and drying climates led to the extinctions of the thermophilic plants or their southward migration to tropical Americas and Asia, or also to Africa but with subsequent extinction in Africa. The West Gondwanan vicariance hypothesis postulates a tropical origin and expansion in southern West Gondwana followed by vicariance from tectonic separation into South America and Africa at ca. 100 Ma [9]. The descendants of West Gondwanan taxa showed the amphi-Pacific tropical distribution through migrations from Africa into tropical Asia followed by extinctions in Africa. In addition, long distance dispersals via birds, wind or ocean currents were suggested for some of these taxa, as there is evidence for their migration over water to the Hawaiian or Polynesian islands in the Pacific Basin or to the Mascarene Islands in the Indian Ocean [2].

Recent biogeographic studies on pantropical taxa suggested only a few good examples (e.g., Strelitziaceae) that have sufficient divergence ages to be explained by the breakup of Gondwana [10]. To our knowledge, the West Gondwanan vicariance hypothesis has not been applied to any taxa showing the amphi-Pacific tropical distribution pattern. Instead, the boreotropics hypothesis has been used to explain the amphi-Pacific tropical distribution in multiple groups (e.g., [10–11]). The estimated divergence times of most other taxa are too young to be explained by the above two hypotheses. Long-distance dispersals have been proposed to play an important role in the formation of their amphi-Pacific tropical distributions (reviewed in [11]). There are only a limited number of biogeographic studies on taxa that show such an amphi-Pacific tropical distribution.

Some genera showing the amphi-Pacific tropical distribution have an extended distribution to the Mascarene Islands, Madagascar, or even eastern tropical Africa [2]. These taxa should be good candidates to test the boreotropics hypothesis and the West Gondwanan vicariance hypothesis involving Africa and/or Madagascar. To our knowledge, there is no biogeographic study on these taxa. *Aphananthe* is a small genus in Cannabaceae demonstrating an amphi-Pacific tropical disjunction with an expanded distribution range to Madagascar. This genus contains only five deciduous to semi-deciduous shrubby or tree species [12–14], with three species from eastern Asia, south and Southeastern Asia and Australia, one in Mexico, and one in Madagascar. *Aphananthe* also has abundant fossil records. The earliest fossil is *Aphananthe cretacea* Knobl. & Mai reported from the Maastrichtian (66–72.1 mya) of Walbeck, Germany [15]. The middle Eocene (48.6–37.2 mya) silicified endocarps of *Aphananthe maii* Manchester were identified from the Nut Beds locality of the Clarno Formation of Oregon, USA [16–17]. The fossil species *Aphananthe tenuicostata* Dorofeev was from the Oligocene (23.0–28.4 mya) of western Siberia [18]. Miocene (16–18 mya) wood fossils were found from Yamagata Prefecture, Japan [19]. Pleistocene (0.0–2.6 mya) pollen fossils were found in Queensland, Australia with a complete pollen record of the last 230 ka from Lynch's Crater, north-eastern Australia. *Aphananthe* thus provides a unique opportunity to explore the evolution of the amphi-Pacific tropical distribution.

*Aphananthe* was strongly supported as member of Cannabaceae by recent molecular studies [20–25]. *Aphananthe* and *Lozanella* were weakly supported to be sister to each other [20]. However most subsequent molecular studies supported *Aphananthe* to be sister to the rest of Cannabaceae [21–25]. Nevertheless, the interspecific relationships of *Aphananthe* remain poorly understood, because only one to three species were sampled in previous studies [20, 23–25]. The origin and evolution of the intercontinental disjunction of *Aphananthe* have never been addressed in previous studies. A fully resolved phylogeny with all species sampled is needed to infer the biogeographic history of this intriguing genus.

We sampled all *Aphananthe* species and employed multiple nuclear and plastid markers to reconstruct their interspecific relationships. Fossil-calibrated divergence time estimates and ancestral geographic range analyses were used to test the alternative hypotheses for the current broad intercontinental disjunctions, with the well confirmed fossils included in the biogeographic analysis.

## Materials and methods

### Taxon sampling

Thirteen individuals representing all five recognized *Aphananthe* species were included in this study (Table 1). Each species (except for *A*. *sakalava* from Madagascar) was sampled with more than one accession. The study sequenced ITS, ETS, *psbA*-*trnH* and *trnL*-*trnF*, and sequences generated from this study are deposited in GenBank (Table 1). Five species from four genera (*Celtis* L., *Gironniera* Gaudichaud-Beaupré, *Lozanella* Greenm., and *Pteroceltis* Maximowicz) representing major clades of Cannabaceae were included as outgroups. More distantly related taxa from Moraceae and Urtiaceae were not sampled as outgroups because of the difficulties in aligning matrices of ETS and ITS.

**Table 1:** Species, voucher, collecting locality, herbarium and GenBank accession number of sampled species.

Species	Voucher, colleting locality, herbarium	GenBank accession number			
		*psbA*-*trnH*	*trnL*-*trnF*	ETS	ITS
*Aphananthe cuspidata* (Blume) Planch.	*YJ WB31-144*, Yunnan, China (KUN)	KR086759	KR086777	KR086725	KR086741
*A*. *cuspidata* (Blume) Planch.	*YMQ 20121016*, Khao Yai, Thailand (KUN)	KR086760	KR086778	KR086726	KR086742
*A*. *monoica* (Hemsl.) J.-F.Leroy	*Ibarra*, *G*. *M*. *2512*, Veracruz, Mexico (UNAM)	KR086763	KR086779	KR086729	KR086745
*A*. *monoica* (Hemsl.) J.-F.Leroy	*Ajcilar*, *G*. *M*. *7624*, Mexico (UNAM)	KR086764	KR086780	KR086730	KR086746
*A*. *philippinensis* Planch.	*NJN 3552*, New South Wales, Australia (KUN)	KR086765	KR086781	KR086731	KR086747
*A*. *philippinensis* Planch.	*Forster*, *P*. *I*. *6657*, Queensland, Australia (L)	KR086766	JN040357	KR086732	KR086748
*A*. *philippinensis* Planch.	*Mabberley*, *D*. *J*. *1785*, Port Moresby, Papua New Guinea (L)	KR086767	KR086782	KR086733	KR086749
*A*. *philippinensis* Planch.	*Craver C*. *A*. *2804453*, Queensland, Australia (MO)	KR086768	KR086783	-	KR086750
*A*. *sakalava* J.-F.Leroy	*Ranirison P*. *5872893*, Antsiranana, Madagascar (MO)	KR086770	KR086784	-	KR086752
*A*. *aspera* (Thunb.) Planch.	*Yi 20111125*, Fukuoka, Japan (KUN)	KR086753	KR086771	KR086719	KR086735
*A*. *aspera* (Thunb.) Planch.	*Chung K-S 110523–28*, South Jeolla, Korea (KUN)	KR086754	KR086772	KR086720	KR086736
*A*. *aspera* (Thunb.) Planch.	*Yi 20111166*, Taiwan, China (KUN)	KR086755	KR086773	KR086721	KR086737
*A*. *aspera* (Thunb.) Planch.	*Yi 20111147*, Shanghai, China (KUN)	KR086756	KR086774	KR086722	KR086738
*Celtis bungeana* Blume	*Gao s*.*n*., Royal Botanic Garden Edinburgh acc. # 19687275	KR086757	KR086775	KR086723	KR086739
*C*. *julianae* C.K.Schneid.	*YMQ 013*, Yunnan, China (KUN)	KR086758	KR086776	KR086724	KR086740
*Gironniera subequalis* Gaudich.	*DNA Barcoding Group B GBOWS 1411*, Yunnan, China (KUN)	KR086761	JN040375	KR086727	KR086743
*Lozanella permollis* Killip & C.V.Morton	*Solomon*, *J*. *C*. *18073*, Cochabamba, Bolivia (U)	KR086762	JN040379	KR086728	KR086744
*Pteroceltis tatarinowii* Maxim.	*Yi 10081*, Guizhou, China (KUN)	KR086769	JN040385	KR086734	KR086751

“–” represents the unavailable sequences.

### DNA extraction, PCR amplification and sequencing

Two nuclear (ETS and ITS) and two plastid (*psbA*-*trnH* and *trnL*-*trnF*) markers were employed in this study. The following primers were used for both amplification and sequencing: “N-nc18S10” and “C26A” [26] for the entire ITS region, or “ITS2” and “ITS5”, “ITS3” and “ITS4” [27] for two separate fragments of the ITS region, respectively; ‘‘18S-IGS” and ‘‘ETS1F” [28–29] for the ETS region; ‘‘psbA-F” and ‘‘trnH-R” [30] for the *psbA*-*trnH* region; primers ‘‘c” and ‘‘f” for the *trnL*-*trnF* region, or primers ‘‘c” and ‘‘d”, ‘‘e” and ‘‘f” for two separate fragments of the *trnL*-*trnF* region, respectively [31].

Total DNA was obtained from silica-gel-dried leaf fragments or herbarium specimens using the CTAB protocol of Doyle and Doyle [32]. Polymerase chain reaction (PCR) amplifications and the sequencing procedure followed those of Yang et al. [25].

### Sequence alignment and phylogenetic analyses

Sequences were initially aligned using Geneious ver. 4.8.2 [33] followed by manual adjustments using Se-Al ver. 2.0 [34]. Phylogenetic analyses were conducted on cpDNA, nrDNA, as well as the combined cpDNA and nrDNA data set, respectively. The data matrices are available at Data Dryad (http://dx.doi.org/10.5061/dryad.9082p).

Topological incongruence between ITS and ETS data sets, as well as between the nrDNA and cpDNA data sets were tested using the incongruence length difference (ILD) test [35] implemented in PAUP* ver. 4.0b10 [36]. For the ILD test, 1000 heuristic searches were carried out after the removal of all invariable characters from the data set [37]. The ILD test between the ITS and ETS partitions estimated a *p* value of 0.71, they were thus combined in the analyses. The phylogenies of the cpDNA and the nrDNA data sets were largely congruent (see S1 and S2 Figs), and the ILD test estimated a *p* value of 0.06 between these two partitions. The nuclear and plastid data sets were thus combined in the following analyses.

Phylogenetic analyses were conducted on the cpDNA data set, the nrDNA data set, and the combined cpDNA and nrDNA data set. For the combined data set, we divided it into three partitions: *psbA-trnH*, *trnL-trnF* and ETS+ITS, and substitution model for each partition was TVM+G, which were identified by the PartitionFinder ver. 1.1.1 [38]. The estimated substitution models were applied in phylogenetic reconstruction using maximum likelihood (ML) and Bayesian inference (BI), and divergence time estimation used a Bayesian approach.

Phylogenetic relationships were inferred with maximum parsimony (MP) as implemented in PAUP* ver.4.0b10 [36], maximum likelihood (ML) as implemented in GARLI ver. 2.0 [39], and Bayesian inference (BI) with MrBayes ver. 3.1.2 [40].

The MP analyses used heuristic searches with 1000 random addition sequence replicates, tree bisection reconnection (TBR) branch swapping, and MULTREES on. All character states were treated as unordered and equally weighted, with gaps as missing data. To evaluate the relative robustness of clades in the MP trees, the bootstrap analysis was performed with 1000 replicates using the same options as above.

For the ML analysis [39], the previously determined partitioning scheme and substitution model for each partition were applied. To obtain statistical support, 1000 bootstrap replicates were conducted with a rapid bootstrapping and subsequent ML search.

For the BI inference, one cold and three incrementally heated Markov chain Monte Carlo (MCMC) chains were run for 20,000,000 generations. Trees were sampled every 100 generations. The previously determined partitioning scheme and substitution model for each partition were applied. MCMC runs were repeated twice to avoid spurious results. Stationarity of the Markov chain was ascertained by plotting and interpreting likelihood values against number of generations in Tracer ver. 1.3 [41]. The first 50,000 trees before stationarity were discarded as burn-in, and the remaining trees were used to construct the majority-rule consensus trees. The average standard deviation of split frequencies between the two runs was 0.001862 and ESS values as computed by Tracer ver. 1.3 [41] were above 200 for two individual MCMC runs. The posterior probabilities (PP) ≥ 0.95 were considered to be significant probability for a clade following that of Alfaro et al. [42].

### Estimation of divergence times

To estimate divergence times between lineages, we employed the combined data matrix. Rate constancy was tested with a likelihood ratio test [43]. Because the data matrix departed from the clock-like evolution, a Bayesian approach as implemented in BEAST ver. 1.7.5 [44] was applied under a log-normal relaxed molecular clock [45] and a Yule pure birth model of speciation to estimate the times of divergence and their credibility intervals. The previously determined partitioning scheme and substitution model for each partition were applied. Posterior distributions of parameters were approximated with two independent MCMC analyses of 20,000,000 generations with a 25% burn-in. Sample from the two runs (which yielded similar results) were combined and convergence of the chains was checked with the program Tracer ver.1.3 [41]. A maximum clade credibility (MCC) tree was produced with Tree Annotator ver. 1.5.4 [44].

Two confident macrofossils were applied as constraints. The oldest fossil species of *Aphananthe*, *A*. *cretacea* with relatively small endocarps reported from the Late Cretaceous (Maastrichtian: 66–72.1 mya) of Germany. The stem age of *Aphananthe* (node A) was thus constrained at 66 mya based on the low boundary age of Maastrichtian. Prior settings for node A were: offset of 66 mya, a log mean of 1.0 (log stdev of 0.5), yielding a prior age distribution with a median age of 68.7 mya and a 97.5th percentile of 73.2 mya. Based on the oldest fossil species of *Celtis*, *Celtis aspera* (Newberry) Manchester, Akhmetiev & Kodrulhas foliage and endocarps from Paleocene (56–64 mya) in eastern Russia as well as the United States and Canada [46], the stem age of *Celtis* (node B) was constrained to 56 mya (the low boundary age of the Paleocene). Prior settings for node node B were: offset of 56 mya, a log mean of 1.0 (log stdev of 0.5), yielding a prior age distribution with a median age of 58.7 mya and a 97.5th percentile of 63.2 mya.

### Biogeographic analyses

We reconstructed the ancestral areas of *Aphananthe* by applying the dispersal-extinction-cladogenesis (DEC) analysis implemented in LAGRANGE [47–48] and the Bayesian Binary MCMC (BBM) analysis implemented in RASP ver. 2.0 [49]. In order to avoid any analytical biases caused by the incomplete sampling of outgroup taxa, we excluded all outgroups. We pruned the maximum clade credibility (MCC) tree of BEAST to include only one individual of each species. To integrate fossil ranges into the reconstructions, three fossil species were placed manually into the newick tree chronogram according to their age. Each fossil was given a short branch length (0.5 Ma), simulating an extinct range. Other fossils were not used in the ancestral area reconstructions (AARs) because they were too young to be assigned to the relevant stem lineage. Six areas of endemism were defined for the biogeographic analyses based on the distribution and phylogeny of the extant and extinct *Aphananthe* species: A, eastern Asia (including China, Japan and Korea); B, southern and southeastern Asia (including India, Indochina, Indonesia, Malaysia and the Philippines); C, North America; D, Australia (including New Guinea); E, Madagascar and F, Europe. For the DEC analysis, we used default settings without imposing any additional expansion or time constraints. For the BBM analysis, we used 1000 MCC trees from the BEAST output. The MCMC chains were run simultaneously for 5,000,000 generations. The state was sampled every 100 generations. Fixed JC + G (Jukes-Cantor + Gamma) were used for the BBM analysis with a null root distribution. The maximum number of areas for this analysis was kept as two.

## Results

### Phylogenetic analyses

The ILD test revealed congruence between ITS and ETS data sets (*p* = 0.71), as well as between the cpDNA and nrDNA data sets (*p* = 0.06). Furthermore, there was no well-supported conflict between cpDNA and nrDNA phylogenies (S1 and S2 Figs). The combined cpDNA and nrDNA data set was applied to reconstruct interspecific relationships of *Aphananthe*.

**Fig 1:**
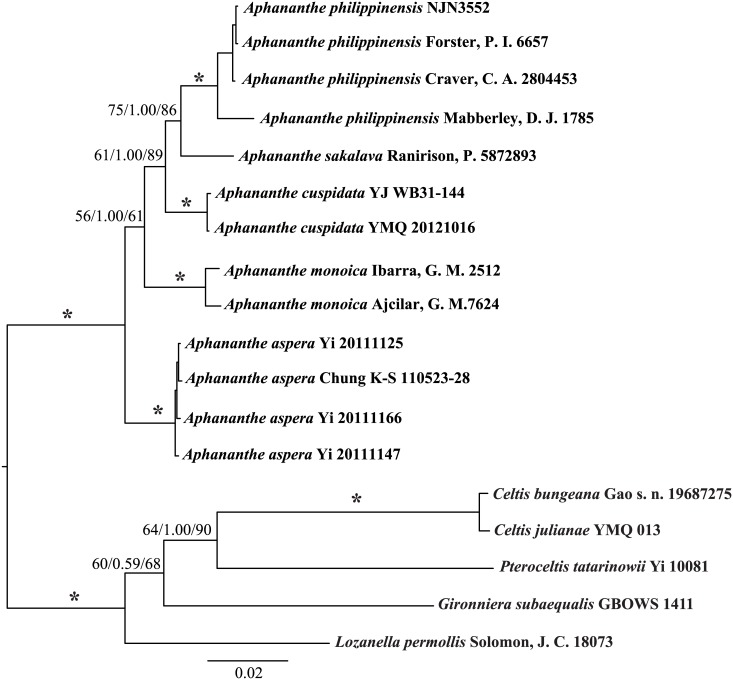
Phylogram obtained from Bayesian inference using the combined cpDNA and nrDNA data set.

The combined chloroplast and nuclear data set included 2579 aligned positions with 349 parsimony-informative characters. The parsimony analysis yielded one most parsimonious tree with 870 steps (CI = 0.856; RI = 0.863, Table 2). The ML analysis and BI yielded similar topologies to the MP tree (Fig 1). Each species was monophyletic with high support except that only one accession of *A*. *sakalva* was sampled. *Aphananthe* was strongly supported as monophyletic (maximum parsimony bootstrap support (MPBS) = 100%; posterior probabilities (PP) = 1.0; maximum likelihood bootstrap support (MLBS) = 100%). The eastern Asian species (*A*. *aspera*) was sister to the clade of the rest of the genus, and the next diverged clade was the Mexican species *A*. *monoica* (MPBS = 56%; PP = 1.00; MLBS = 61%). The clade of *A*. *philippinensis* and *A*. *sakalava* was moderately supported (MPBS = 61%; PP = 1.00; MLBS = 89%) and was sister to *A*. *cuspidata* (MPBS = 75%; PP = 1.00; MLBS = 86%).

**Table 2:** Characteristics of individual and the combined data sets of *Aphananthe*.

Gene region	No. of taxa	Aligned length	Variable sites	PI sites
*psbA-trnH*	18	590	111	48
*trnL-trnF*	18	953	123	67
ETS	15	311	133	92
ITS	17	725	237	142
cpDNA data set	17	1534	234	115
nrDNA data set	18	1036	370	234
combined cpDNA and nrDNA data set	18	2579	604	349

PI sites = parsimony-informative sites.

Bootstrap values (%) of MP, the PP values of BI analysis, and bootstrap values of ML analysis are shown above the branch (MPBS/PP/MLBS, asterisks indicate 100% support by MPBS/PP/MLBS values).

### Divergence time estimations and biogeographic inference

**Fig 2:**
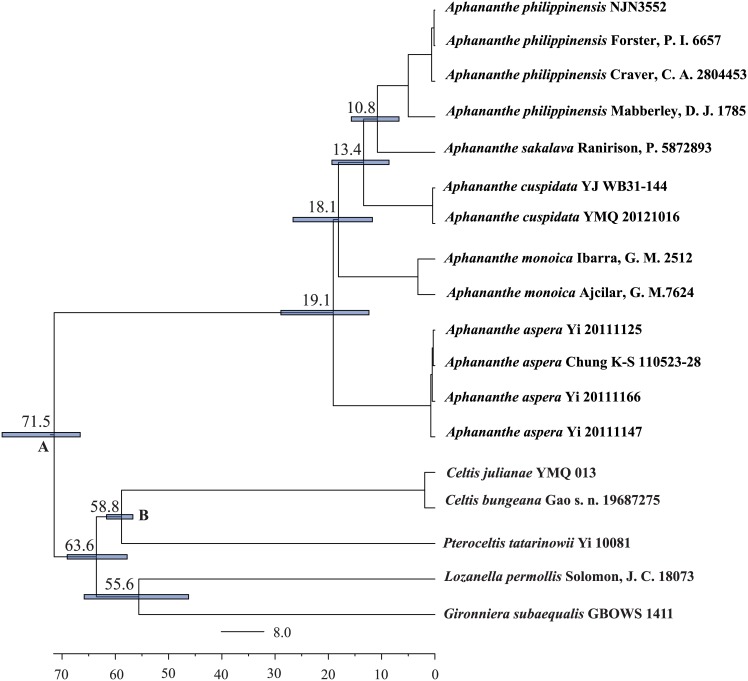
The maximum clade credibility chronogram of *Aphananthe* and its relatives inferred by the BEAST analysis. Node A was constrained to 66 mya and node B was constrained to 56 mya.

The stem age of *Aphananthe* was estimated to be 71.5mya (95% HPD: 66.6–81.3 mya); and the crown age was estimated to be 19.1 mya (95% HPD: 12.4–28.9 mya) (Fig 2).

**Fig 3:**
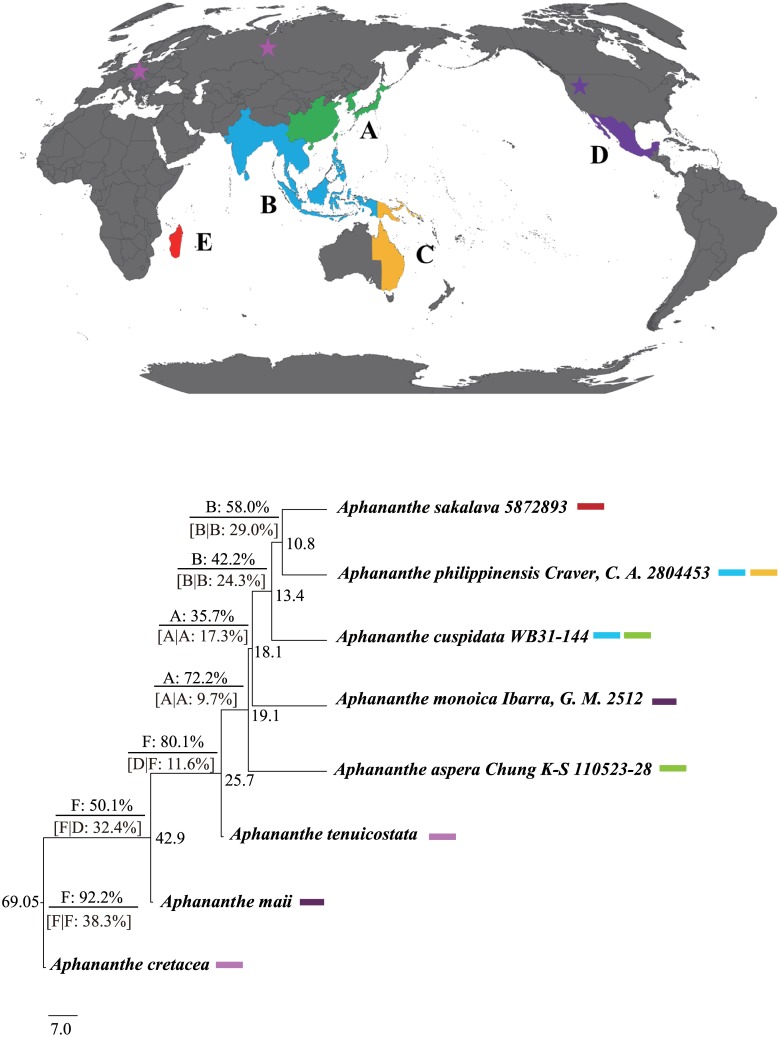
Results of the biogeographic reconstruction for *Aphananthe* estimated using Bayesian Binary MCMC and the DEC analyses.

The results of the DEC and BBM analyses were largely similar (Fig 3), which suggested Europe as the ancestral area for *Aphananthe*, and eastern Asia for extant clade of *Aphananthe* species. *Aphananthe* migrated from eastern Asia to North America at 18.1 mya (95% HPD: 11.7–26.6 mya) and southern and southeastern Asia at 13.4 mya (95% HPD: 8.6–19.3 mya), and from southern and southeastern Asia to Australia and Madagascar at 10.8 mya (95% HPD: 6.7–15.7 mya).

The most likely ancestral ranges of nodes with frequency of occurrence inferred by the Bayesian Binary MCMC analysis are shown above branches, those by the DEC analysis below branches. For results of the DEC analysis, a slash indicates the split of areas in two daughter lineages, i.e., left/right, where “left” and “right” are the ranges inherited by each descendant branch. The distribution ranges were divided into six areas for both analyses: A, eastern Asia (including China, Japan and Korea); B, southern and southeastern Asia (including India, Indochina, Indonesia, Malaysia and the Philippines); C, North America; D, Australia (including New Guinea), E, M**a**dagascar; and F, Europe. Stars indicate the fossil records, the pink star indicates late Cretaceous and Oligocene fossils in Europe, and the purple star indicates the middle Eocene fossil in North America. The background map was downloaded from http://www.naturalearthdata.com/; the figure is similar to the original one but not identical to it, and therefore it is for illustrative purposes only.

## Discussion

### Phylogenetic relationships in *Aphananthe*

Our phylogenetic analyses strongly support the monophyly of *Aphananthe* (Fig 1). All *Aphananthe* species share a few indels in the *trnL-trnF* data: three deletions and two insertions in *trnL-trnF* (a 5-bp deletion at position 110–114bp; an 88-bp deletion at position 664–751bp; a 7-bp insertion at position 752–758bp; and an 82-bp insertion at position 795–876bp), which further support the monophyly of *Aphananthe*. *Aphananthe* was considered to be isolated from other members of Cannabaceae based on morphology and molecular evidence. *Aphananthe* possesses asymmetrical ovules (symmetrical ovules in other Cannabaceae) and flavonol production (the only genus producing flavonol in Cannabaceae) [50]. *Aphananthe* is the only genus of Cannabaceae which has x = 13 [51]. A distinct pattern of diversity in seed coat morphology, as well as the occurrence of a unique derived seed coat feature (i.e., the raised middle part of exotestal cells in *Aphananthe aspera*), may also suggest that *Aphananthe* is in an isolated lineage in Cannabaceae [52].

The monophyly of each species was strongly supported and their relationships were resolved and well supported (Fig 1). *Aphananthe* species are also easily distinguishable by morphological characters: *A*. *aspera* possesses trinerved venation (rather than pinnate venation in other species); *A*. *cuspidata* is characterized by its large leaf blade (5-)10-15 × (2-) 3–5 (-7) cm, usually with entire leaf margin, and large fruits 1.3–2 × 0.7–1.2 cm; *A*. *philippinensis* can be distinguished from other species by its relatively small leaves (4–6 cm × 1.5–3 cm vs. 5–15 cm × 3–7 cm in other species), and its sharply toothed leaf margin; *A*. *sakalava* is characterized by its cymose female inflorescences of 2–3 axillary flowers; and the leaves of *A*. *monoica* have the highest number of secondary veins (18–20 pairs vs. 6–14 pairs in other species).

### The early diversification in the Northern Hemisphere

Previous studies on the diversification of lineages have shown that a long stem leading to a crown of short branches may indicate major early extinctions [53–54]. Multiple taxa including Orontioideae of Araceae [55], *Hamamelis* L. [56], and *Hedyosmum* Sw. [57] have shown such a phylogenetic pattern. A similar pattern was detected in *Aphananthe*, which contains a remarkable temporal difference (52.4 million years) between the stem lineage age (71.5 mya, with 95% HPD: 66.6–81.3 mya) and the crown age of the extant species (19.1 mya, with 95% HPD: 12.4–28.9 mya). Furthermore, the fossil records indicate that *Aphananthe* was widely distributed in the middle to high latitudes of the Northern Hemisphere including Europe, western Asia and North America during the early Tertiary until the Oligocene [15–16, 18]. Climates were warm enough to support the thermophilic vegetation at the high latitudes in the Northern Hemisphere during the Eocene [4–5], and many fossils of thermophilic taxa were recovered across this region [3, 5, 58–59]. The phylogenetic and dating results as well as fossil data suggest that *Aphananthe* was part of the high latitude thermophilic elements during the early Tertiary, and it subsequently experienced extinctions from Europe and North America in the middle Tertiary when a dramatic cooling of climates extirpated many evergreen plant lineages that were once part of the Holarctic boreotropical flora [5]. Many Northern Hemisphere lineages experienced similar histories [56, 60, 61].

### Formation of amphi-Pacific tropical distribution

The West Gondwanan vicariance hypothesis could be confidently excluded as an explanation for the amphi-Pacific tropical distribution of *Aphananthe*. Our ancestral range analyses strongly support a Northern Hemisphere boreotropical origin for *Aphananthe*, and eastern Asia as the ancestral area for the extant species. Although *Aphananthe* has a relatively old stem age (71.5 mya), the estimated crown age (19.1 mya) of the extant species is much younger than that expected for a West Gondwanan origin (> c. 84 Ma). The North American and Madagascan lineages were inferred to have migrated from eastern Asia, southern and southeastern Asia instead of their Northern Hemisphere regions. Furthermore, both migrations occurred during late Tertiary, which were too young to be explained by the boreotropics hypothesis. Long-distance dispersals from eastern Asia during Miocene may be the best hypothesis to account for the current amphi-Pacific tropical distribution of this genus.

This genus migrated into North America by the early Miocene (18.1 mya, 95% HPD: 11.7–26.6 mya). This migration into North America was perhaps through the Bering land bridge [61]. Furthermore, we could not exclude the possibility of a long-distance dispersal via birds. The Miocene was an important period for the migration of plant taxa between the eastern Asian and North America [4, 7, 62–65].

The region of eastern Asia to Australia harbors three of the five *Aphananthe* species and is the diversification center of the genus. *Aphananthe aspera* has the northernmost distribution in eastern Asia (China, Japan, Korea, to the northern Vietnam). *Aphananthe cuspidata* has a more southern distribution in southern and southeastern Asia (from southern China to India, Malaysia, to Indonesia). *Aphananthe philippinensis* is distributed in the Philippines, New Guinea and Australia. *Aphananthe aspera* was resolved to be the first diverged clade of the genus at 19.1 mya (95% HPD: 12.4–28.9 mya, Fig 2). *Aphananthe cuspidata* represents the second diverged clade (13.4 mya, 95% HPD: 8.6–19.3 mya) within this region, and *A*. *philippinens* is the third diverged lineage (10.8 mya, 95% HPD: 6.7–15.7 mya) within this region. *Aphananthe* seems to have experienced species diversification following the southward and southeastward dispersal from eastern Asia to India, the Malesian region, and Indochina (i.e., the regions extends from southern Thailand through Malaysia, Singapore, Indonesia, eastern Timor and the Philippines, to Papua New Guinea and the Solomon Islands via southeastern Asia, or the mountain ranges of Taiwan and the Philippines), and Australia. Immigration of the continental Asian taxa is the major resources of flora of Malesia [66–68]. Recent molecular phylogenetic and biogeographic studies also supported the major north-to-southeast dispersal of taxa in this region [69–74].

Species of *Aphananthe* most likely dispersed eastwards across the Wallace’s line into the Wallacea, New Guinea and Australia in the middle Miocene. The “west-to-east” dispersal was inferred from other plant taxa of different families, e.g., *Pseuduvaria* Miq. (Annonaceae) [72], *Alocasia* (Schott) G. Don (Araceae) [75], *Begonia* L. (Begoniaceae) [76] and tribe Millettieae (Fabaceae) [77]. This west-to-eastward dispersal is consistent with the geologic history of the region. The land of the western Malesia remained emerged throughout the Cenozoic, while the emergence of the land masses east of the Wallace’s line including the Wallacea, Sulawesi, New Guinea and a series of volcanic islands along the Sunda Arc, the Banda Arc, and the Halmahera Arc connecting these region from the late Miocene onwards, supplying a potential channel for dispersal by island-hopping between western and eastern Malesia [78–79]. The niche pre-emption following the formation of new landmass in the eastern Malesia [80–81] may have facilitated the similar west-to-east dispersal pattern observed in various taxa with different life forms, generation times and dispersal capabilities [76, 79, 82].

### Biogeographic disjunction between Asia and Madagascar

Four major hypotheses have been proposed to explain the disjunctions across paleotropical regions around the Indian Ocean Basin: (1) the “out-of-India” hypothesis, i.e., rafting of biota of Gondwanan origin by the Indian plate and subsequent dispersal to Asia during the Cenozoic [83–85]; (2) the “boreotropical migration” hypothesis, i.e., dispersal through a northern mid-latitude corridor of extensive boreotropical forests during the Paleocene and Eocene [3, 86–87]; (3) the overland migration across Arabia during a warm phase in the early to middle Miocene [88]; and (4) the transoceanic long-distance dispersal [89–90]. *Aphananthe sakalava* from Madagascar was inferred to be dispersed from southern and southeastern Asia at around 10.8 mya (6.7–15.7 mya) or less. The Indian landmass separated from Madagascar during the mid-late Cretaceous (90–85 mya), and the collision of the Indian subcontinent with the Eurasian continent was estimated at ca. 50 mya [83] to 35 mya [91, 92], which is much earlier than the estimated dispersal age of *Aphananthe* from Asia to Madagascar. Furthermore, the dispersal direction is inferred from southern and southeastern Asia to Madagascar instead of the reverse. The “out-of-India” hypothesis thus can not be applied to explain the disjunction between Asia to Madagascar. The “boreotropical migration” hypothesis also seems unlikely, because this corridor was disrupted following climatic deterioration in the late Eocene [3, 86–87, 93]. The estimated age is too young to be consistent with this hypothesis. Overland migration between Africa and Eurasia through Arabia has been hypothesized for some tropical plant taxa [88, 94]. Africa and Eurasia were connected during the early to middle Miocene [95], which coincided with a warm phase with the peak at the Middle Miocene Climatic Optimum during 17–15 mya [93]. The estimated dispersal time is later than this climate optimum, and the extant *Aphananthe* species are absent from Arabia and continental Africa. There are no reported fossils there, and extinctions from both regions are required to apply this hypothesis to explain this disjunction, making this hypothesis less likely. The long-distance dispersal seems the most likely hypothesis for this disjunct pattern in *Aphananthe*. There are no reports of the adaptation of *Aphananthe* or other Cannabaceae fruits or seeds to hydrochory, and *Aphananthe* species are absent from sea shores. *Aphananthe* thus most likely arrived in Madagascar through the long-distance dispersal by birds, as fruits of *Aphananthe* are eaten and dispersed by many kinds of birds [96–98]. This dispersal may have been promoted by two factors. First, the Indian winter monsoon winds blow from the Indian subcontinent towards the Madagascar region [99–100]. Second, sea level has fluctuated in the recent geologic past, and a chain of islands stretched between the granitic Seychelles, Mascarenes and India. Such stepping-stones may have provided a channel for communication of plants and animals between Asia and Madagascar [101–102]. This scenario has been applied to explain dispersals from Asia to Madagascar in a few recently studied taxa [90, 103–104]. Recent molecular studies overwhelmingly favor long-distance dispersals from southeastern Asia to Africa [88, 105–108], rather than the rare dispersals from Africa to Asia [109].

## Conclusions

Our analyses largely resolve the phylogenetic relationships among *Aphananthe* species. The temporal and spatial diversification patterns of *Aphananthe* were explored with respect to the paleoenvironmental changes. A long stem (52.4 million years) of *Aphananthe* indicated the ancient origin and relatively recent diversification of extant species. Fossil records indicate that *Aphananthe* was widely distributed in Northern Hemisphere during the early Tertiary until the Oligocene, and experienced major extinctions from western Eurasia and North America during the middle Tertiary. The oldest fossil of *Aphananthe* was from Europe, and eastern Asia was inferred to be ancestral region of the extant clade of *Aphananthe* species. The broad intercontinental disjunction across eastern Asia, southern and southeastern Asia, Australia, North America and Madagascar is due to migration via the Bering land bridge and the long-distance dispersals via birds during the Miocene. Significant climatic and geologic changes have shaped the species diversification and distribution pattern of this biogeographically unique genus *Aphananthe*.

## Supporting information

S1 Fig Phylogram obtained from Bayesian inference using the combined cpDNA data set. Bootstrap values (%) of MP, the PP values of BI analysis, and bootstrap values of ML analysis are shown above the branch (MPBS/PP/MLBS, and asterisks indicate 100% MPBS/PP/MLBS values, dashes indicate the MPBS/PP/MLBS values are less than 50%).
(EPS)

S2 Fig Phylogram obtained from Bayesian inference using the combined nrDNA data set. Bootstrap values (%) of MP, the PP values of BI analysis, and bootstrap values of ML analysis are shown above the branch (MPBS/PP/MLBS, and asterisks indicate 100% MPBS/PP/MLBS values, dashes indicate the MPBS/PP/MLBS values are less than 50%).
(EPS)
